# Prediction Method for High-Speed Laser Cladding Coating Quality Based on Random Forest and AdaBoost Regression Analysis

**DOI:** 10.3390/ma17061266

**Published:** 2024-03-08

**Authors:** Yifei Xv, Yaoning Sun, Yuhang Zhang

**Affiliations:** 1 School of Mechanical Engineering, Xinjiang University, Urumqi 830017, China; xyf124@stu.xju.edu.cn; 2 Institute of New Materials, Guangdong Academy of Sciences, National Engineering Laboratory for Modern Materials Surface Engineering Technology, Guangdong Provincial Key Laboratory of Modern Surface Engineering Technology, Guangzhou 510651, China

**Keywords:** high-speed laser cladding, random forest, AdaBoost, regression analysis, coating quality prediction

## Abstract

The initial melting quality of a high-speed laser cladding layer has an important impact on its post-treatment and practical application. In this study, based on the repair of hydraulic support columns of coal mining machines, the influence of high-speed laser cladding process parameters on the quality of Fe-Cr-Ni alloy coatings was investigated to realize the accurate prediction of coating quality. The Taguchi orthogonal method was used to design the L_25_(5^6^) test. The prediction models of the relationship between the cladding process and the coating quality were established using the Random Forest (RF) and AdaBoost (Adaptive Boosting, AB) algorithms, respectively. Then, the prediction accuracy of the two models was compared, and the process parameter features were screened for importance evaluation. The results show that the AB prediction model is more accurate than the RF prediction model and more sensitive to abnormal data. The importance evaluation based on the AdaBoost model shows that the scanning speed has a great influence on the height and surface roughness of the coating. On the other hand, the overlap rate is the most important factor in controlling the dilution ratio and near-surface grain size of high-speed laser melting coatings. In addition, the micro-hardness of the coating and the thermal effect of the substrate can be effectively enhanced by adjusting the laser power and scanning speed. Finally, it was verified that the AB prediction model could accurately estimate the quality indexes of the coating with a prediction error less than 6%. The results show that it is feasible to predict the quality of high-speed laser cladding with the AB algorithm. It provides a basis for the adjustment of process parameters in the subsequent quality control process of cladding.

## 1. Introduction

The coating prepared via laser cladding technology can not only ensure the bonding strength but also meet the requirements of protective properties [1,2]. Thus, in recent years, as a representative of green manufacturing, laser cladding has been increasingly used in the preparation of protective coatings. However, this process has the disadvantages of low efficiency, insufficient forming quality and accuracy, and the subsequent need for two or even multiple procedures to meet the requirements of use. Alongside that, the cladding layer has high crack sensitivity, and the crack control in the forming process is also very difficult [3,4]. To solve these problems, the German Fraunhofer Institute of Laser Technology invented extreme-high-speed laser cladding (EHLA) [5]. EHLA replaces the traditional electroplating process as an advanced green manufacturing technology and has broad application prospects. Currently, high-speed laser cladding has been applied to the oil drilling and production part repair industry to address the issues that traditional laser cladding has a great impact on the heat of the substrate and the processing efficiency is low. As a new generation of processing technology, its working principle and heat transfer mode are very different from traditional cladding technology. Owing to the shielding effect of the powder material on the laser energy and the two-stage melting process before/after entering the melt pool, the substrate has a low heat input, the coating dilution ratio is smaller than 1/4 of traditional cladding, and the surface roughness is reduced to less than 1/10 of traditional cladding [6]. Additionally, it has a 100–250 times higher cladding speed than the traditional cladding, which leads to the fine grain of the coating, thus allowing for the improvement of all aspects of the coating performance [7,8].

High-speed laser cladding is a complex metallurgical process, and the stability of the entire process is affected by many factors such as process parameters, equipment conditions and even materials [9]. In the cladding process, the laser beam, powder material and substrate interact, and the cladding parameters (such as laser power, scanning speed, overlap ratio and powder feeding rate) affect each other. Given this, constructing a model to predict the cladding quality is the main way to achieve the quality control of high-speed laser cladding coating. Generally, there are three main methods to construct the relationship model between input machining parameters and output machining results: the finite element method, the phenomenology method, and the machine learning method, to investigate the relationship between process parameters and their responses in the laser cladding process [10,11]. The finite element method [12,13] normally constructs models through physical driving to make predictions. Kumar et al. [14] proposed and solved a set of dimensionless transport equations to study the laser metal deposition process, and they calculated the deposition trajectory geometry, dilution ratio, and maximum molten pool temperature. Chai et al. [15] investigated the temperature field of Ni316AA formed by laser cladding based on the balance of gravitational potential energy and interfacial free energy of molten metal, and they calculated the profile of cladding orbit using an improved droplet formation method. Nevertheless, the current physical drive method cannot simulate the entire cladding process in a short time, so it fails to predict the machining result quickly. The phenomenological method is a type of analysis method based on multivariate mathematical statistics. It connects important controllable process parameters with required geometric features through mathematical relations [16,17,18]. It is mainly designed and developed by factor design, Taguchi design, central composite design (CCD), and response surface method (RSM) [19]. Menghani [20] employed a full factorial design approach to briefly explore the overlap rate, microhardness and microstructure of individual coatings while determining optimal cladding conditions through multi-response optimization. Khorram [21,22], respectively, utilizing response surface methodology and central composite design for cladding 75Cr_3_C_2_ + 25(80Ni20Cr) coating, investigated its influence on geometric parameters (width, height, and cladding angle), dilution rate, and hardness. The mathematical statistical analysis process of the phenomenological method usually requires basic experiments. Its statistical analysis is completely based on experimental data, and the accuracy of regression is closely related to the number of experimental samples. Machine learning (ML) is a data-driven approach that directly mines the relationships between input and output data. The use of this relationship for classification and prediction can greatly simplify the forecasting model, improve the forecasting efficiency, and shorten the forecasting time [23]. Le et al. [24] used backpropagation neural network (BPNN) and particle swarm optimization (PSO) algorithms to establish a prediction model between process parameters and laser cladding coating morphology. To guarantee coating molding quality, Deng et al. [25] established the back-propagation neural network (BPNN) and quantum-behaved particle swarm optimization (QPSO) neural network prediction model to obtain the mapping relationship between the laser cladding preparation process parameters and Ti (C, N) ceramic coating microhardness. Chen et al. [26] constructed a prediction model of coating quality characteristics based on support vector machine (SVM) to correctly describe the relationship between cladding process parameters (input variables) and coating quality characteristics (output characteristics). Zhao et al. [27] developed a laser cladding parameter optimization method based on RSM and NSGA-II algorithms to obtain the best process parameters of laser cladding TC4 alloy powder. Gao et al. [28] utilized 316 models of a backpropagation neural network (BPNN), random forest (RF) algorithm and response surface method (RSM) to establish the predictive relationship between deposition input parameters and machining state parameters, geometric morphology and mechanical property parameters. The prediction time of data-driven methods is greatly reduced compared with physically driven methods.

In this study, 45# steel, which is commonly used for hydraulic pillars in the coal mining industry, is used as the matrix material. The Fe-Cr-Ni alloy powder, which has good wear resistance and weak acid corrosion resistance at room temperature below 500, and has a wide range of raw materials and cheap price, is chosen as the cladding material. Two models were constructed based on RF and AB algorithms for the coatings prepared using high-speed laser cladding technology, respectively. Firstly, the L_25_(5^6^) experiment was designed based on the Taguchi orthogonal method, and the combination of different process parameters was taken as the research object. Secondly, RF and AB models were constructed for training. Process parameters such as laser power (P), scanning speed (Ss), overlap ratio (Or) and powder feeding rate (Vp) were taken as the input, and the response values such as cladding layer height (H), molten pool depth (D), dilution ratio (η), grain size (Ds), surface roughness (Ra) and microhardness (HV0.2) were taken as the output. After the model training was completed, the process parameters of the training group were again input into the model for prediction, and the preliminary prediction performance verification of the predicted model was carried out. Then, the accuracy of the two established prediction models was compared, and the characteristics of the process parameters were screened for the importance assessment. Finally, the AB prediction model was further used to predict the cladding layer height (H), molten pool depth (D), dilution ratio (η), grain size (Ds), surface roughness (Ra) and microhardness (HV0.2) of the coating under six combinations of new process parameters.

## 2. Experimental Design

### 2.1. Experimental Equipment and Materials

**Figure 1:**
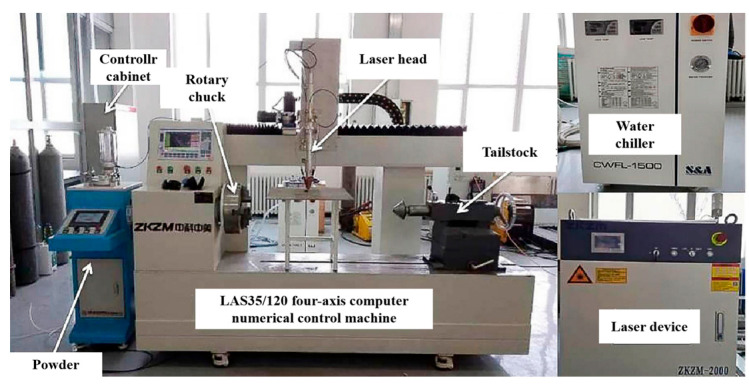
ZKZM-2000 fiber optic high-speed laser system.

The test system adopts the ZKZM-2000 fiber optic high-speed laser system (Zhongke Zhongmei Laser Technology Co., Ltd., Zhangjiagang, China). The experimental equipment is illustrated in Figure 1, and the equipment parameters are listed in Table 1. The base material is ASTM 1045 plate, with a size of 70 mm × 150 mm × 8 mm, and it is the common material for the hydraulic support column of coal machines. The powder material is an Fe-Cr-Ni-based alloy powder (the powder particle size ranges between 35 and 53 μm, Nanjing Zhongke Yuchen Co., Ltd., Nanjing, China). The composition of the Fe-Cr-Ni-based alloy powders is presented in Table 2. Before the test, the surface of the substrate is ground with 400 grit sandpaper and then wiped with alcohol to remove the oil; subsequently, it is dried for use in a drying oven, with a holding temperature of 100 °C and a holding time of 2 h.

The ZKZM-2000 fiber high-speed laser system is used in the experiment. The high-speed laser cladding process uses +15 mm defocusing, and the spot size is 1.2 mm. The powder is fed by a powder feeder with a high-precision coaxial nozzle, which ensures a very small focused powder point (1.2 mm) even at a high powder feed speed. The shielding gas and powder gas are argon gas (purity 99.99%).

### 2.2. Experimental Principle

**Figure 2:**
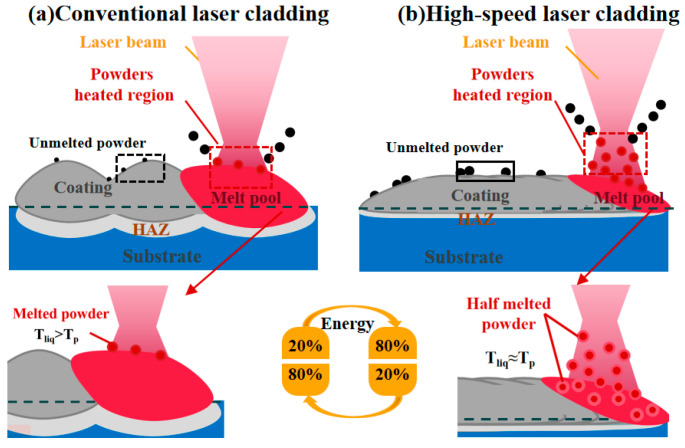
The schematic of the process principle: (**a**) conventional laser cladding; (**b**) high-speed laser cladding.

The differences between the principles of high-speed laser cladding and traditional laser cladding processes are demonstrated in Figure 2 (the red area represents the coating during the melting process, the dark gray area represents the solidified fused coating, the light gray area represents the heat affected zone (HAZ) of the substrate during the melting process, and the blue area represents the substrate). The main differences lie in the way the powder is melted and the way the molten pool is formed. As shown in Figure 2a, in traditional laser cladding, most of the energy of the high-energy laser beam is acted on the matrix melting pool, and only a small amount of energy is acted on the powder particles. Then, the powder particles are fed into the molten pool via the combined action of the powder gas and gravity. Because of this, the temperature of the molten pool is higher than that of the powder particle T_p_. As shown in Figure 2b, in high-speed laser cladding, the high-energy laser beam is shielded by powder. Most of the energy is acted on the powder particles to heat the powder to near its melting point. The remaining small amount of energy is acted on the substrate to form an extremely shallow molten pool. The semi-molten powder particles are sprayed into the molten pool at a high speed, and an extremely thin metallurgical layer is formed after short contact. The temperature of the molten pool is almost consistent with that of powder particles (T_liq_ ≈ T_p_) [8]. Thus, compared with the traditional laser cladding substrate, the high-speed laser cladding bath absorbs less energy, so the bath is shallower and the zone affected by heat is smaller.

### 2.3. Experimental Design

To construct the prediction model of high-speed laser cladding parameters and cladding layer quality, certain experiments are required as a training group. According to the existing research, laser power (P), scanning speed (Ss), overlap ratio (Or) and powder feeding rate (Vp) are the most important process parameters that affect the quality of the cladding layer, so only these four parameters need to be changed in the experimental design. For regression models, the more experimental results can be referred to during training, the better the fitting effect of the model, but the higher the complexity of the experiment. Therefore, L_25_(5^6^) experiments were designed in this study based on the Taguchi orthogonal, and the statistical results were taken as training sets for the RF model and AB model.

### 2.4. Experimental Method

**Figure 3:**
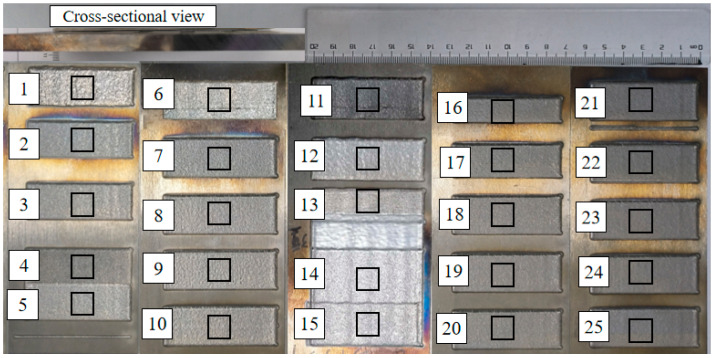
The macroscopic morphology of high-speed laser cladding coating.

The coating was prepared via high-speed laser cladding. The specimen was cut perpendicular to the scanning direction, with a cutting size of 10 mm × 10 mm × 8 mm. The metallographic sample was then etched with aqua regia (=3:1) for 30 s. The height of the cladding layer and the depth of the molten pool of the cross-section were observed with the VS200-500U optical microscope (Shenzhen AOSVI Microoptical Instrument Co., Ltd., Shenzhen, China) (3 position data were taken for each sample), and the coating dilution ratio was calculated according to Equation (1). On the other hand, the surface roughness was measured by the VHX-6000 ultra-depth field microsystem (Keens (China) Co., Ltd., Shanghai, China). The microhardness of the coating was measured by the HVS-1000B Vickers microhardness tester (Dongguan Zhongte Precision Instrument Technology Co., Ltd., Dongguan, China) (the loading load was 200 g, the loading time was 15 s, and the hardness test was conducted three times at the same height of 50 μm from the surface, and the average value was taken as the hardness value of the position). Alongside that, the microstructure of the coating was observed by the LEO-1430VP scanning electron microscope (SEM) (LEO Electron Microscopy Ltd., Jena, Germany). Additionally, the grain size of the coating near the surface, magnified 5000 times, was measured using ImageJ software (1.8.0.345). The macroscopic morphology of the sample is illustrated in Figure 3, the factors and levels of cladding experiment design are shown in Table 3, and the response results are presented in Table 4.

(1) $$\eta \approx \frac{\sum_{i=1}^{n}\frac{D_{i}}{D_{i}\;+\;H_{i}}}{n}$$

where *D_i_* and *H_i_* represent the depth of the molten pool and the coating height on the *i*-th channel, respectively; *n* represents the total number of molten channels.

## 3. Model for Predicting the Quality of the Cladding Layer

The most intuitive way to evaluate the model is to measure its prediction accuracy and prediction time. RF and AB are non-parametric regression models with a good prediction effect, so there is no need to test the hypothesis conditions such as normality and independence of variables, nor consider the collinear problem of multiple variables and adjust the parameters multiple times. Also, the importance of each parameter to the characteristics of the response can be measured. This feature facilitates an in-depth understanding of the high-speed laser cladding process.

### 3.1. Establishment of Prediction Model

**Figure 4:**
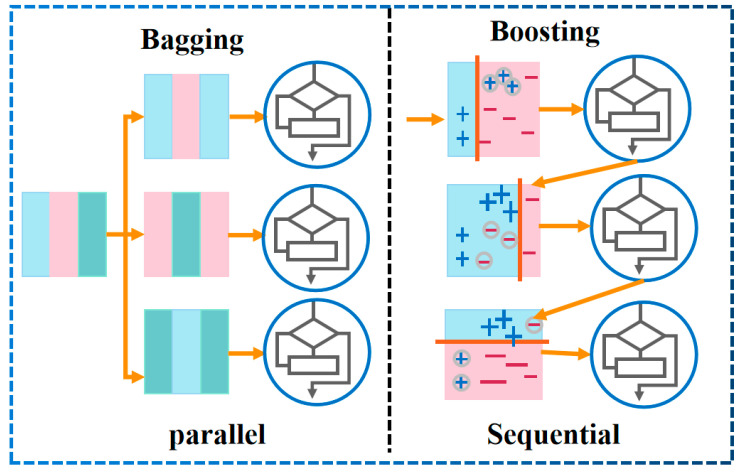
Strategy comparison between RF and AB algorithms.

Ensemble learning is mainly divided into the bagging algorithm and the boosting algorithm, and their major differences are demonstrated in Figure 4. RF, first proposed by Breiman [29], is a collection of classification and regression trees. It is a modified algorithm based on the Bagging strategy. In the process of generating each decision tree, the samples and characteristic variables of the training dataset are randomly sampled, and each decision tree will generate rules and judgment values according to its attributes. Then, the forest realizes the regression of the random forest algorithm by integrating the rules and judgment values of all decision trees [30]. RFs generally prevent overfitting by reducing the complexity of each tree and the number of features used to build the decision tree. AB is a typical Boosting algorithm. Its operation principle is similar to the human learning process: each learning process will gain the experience of the previous one and correct the previous mistake. The strong classifier synthesized using this method retains the advantages of the weak classifiers and weakens their disadvantages. When the AB algorithm runs, it first assigns the same weight to each weak classifier, then updates the weight of each classifier according to the result of the weak classifier, and synthesizes a strong classifier according to the weight obtained in the last iteration [31]. The algorithm has a simple structure and strong robustness, can handle continuous and discrete values, and possesses great advantages in reducing bias and improving the accuracy of deep learning.

A high-speed laser cladding coating quality prediction model was constructed based on RF and AB algorithms. The model building process is as follows:

#### 3.1.1. The RF Model

1. A training set with n samples was selected from the high-speed laser cladding parameter combination sample set using the Bootstrap method. The selected samples were used to train a decision tree as the sample of the decision tree node.
2. m features were randomly selected from the 6 features of the sample, satisfying the condition m << 6. Then, 1 feature was chosen from the m features as the splitting feature of the node; steps 1 to 2 were repeated 100 times, where 100 is the number of decision trees in the RF.
3. The trained RF was used to predict the test sample, and the prediction result was obtained using the voting method.

**Figure 5:**
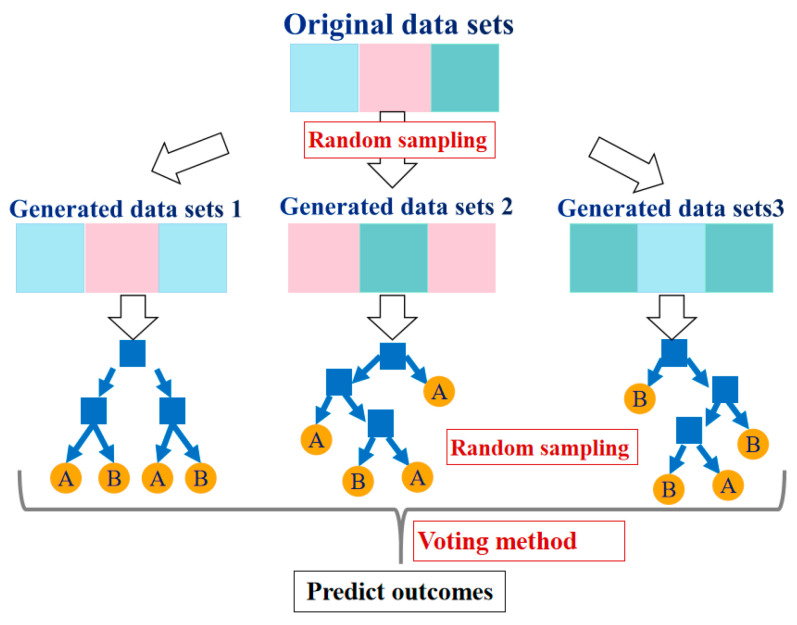
The prediction flow of the RF model.

The construction process of the RF model is shown in Figure 5.

#### 3.1.2. The AB Model

1. m samples were selected from the 25 sample sets of high-speed laser cladding as a training set D, D = ((*x*_1_, *y*_1_), (*x*_2_, *y*_2_), …, (*x_m_, y_m_*)).
2. A sampling weight *D*_1_(*x*) = *w_j_* was assigned to each training sample, with the initial *w_j_* = 1/*m*, *i* = 1. A training set was generated by sampling with replaceability, with an equal volume and weight. A weak learner $h_{t}\left(x\right)$ was assigned for each training round, and the weight of the weak learner $\alpha_{t}$ was calculated.
3. Weak learners were combined into a strong learner: $H\left(x\right)=\;\mathrm{sign}(\overset{T}{\underset{t1}{\sum }}\alpha_{t}h_{t}\left(x\right)$).

**Figure 6:**
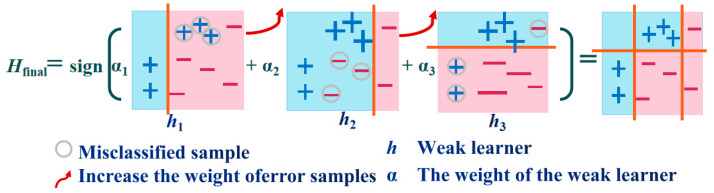
The prediction flow of the AB model.

The prediction flow of the AB model is shown in Figure 6.

RF and AB models were trained with 25 sets of experiments. Given the need to predict cladding coating quality under different process parameters, the design input (*Xi*) is

*X_i_* = [*P_i_, Ss_i_, Or_i_, Vp_i_*] (*i* = 1, …, *n*) (2)

where *P_i_* stands for the laser power, *Ss_i_* stands for the scanning speed, *Or_i_* stands for the overlap ratio and *Vp_i_* stands for the powder feeding rate.

For each set of defined inputs, there is a corresponding set of output responses:

*Y_i_ =* [*H_i_, D_i_, η_i_, Ds_i_, Ra_i_, Hv_i_*]     (*i* = 1, …, *n*) (3)

where *H_i_*, *D_i_*, *η_i_*, *Ds_i_*, *Ra_i_* and *Hv_i_* represent the cladding layer height, molten pool depth, dilution ratio, grain size, surface roughness and microhardness, respectively.

### 3.2. Tests and Results

**Figure 7:**
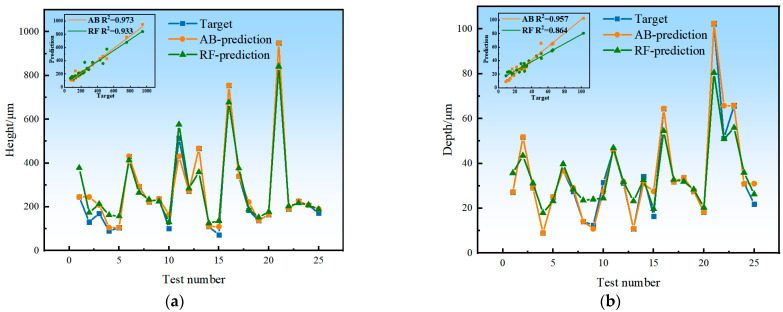

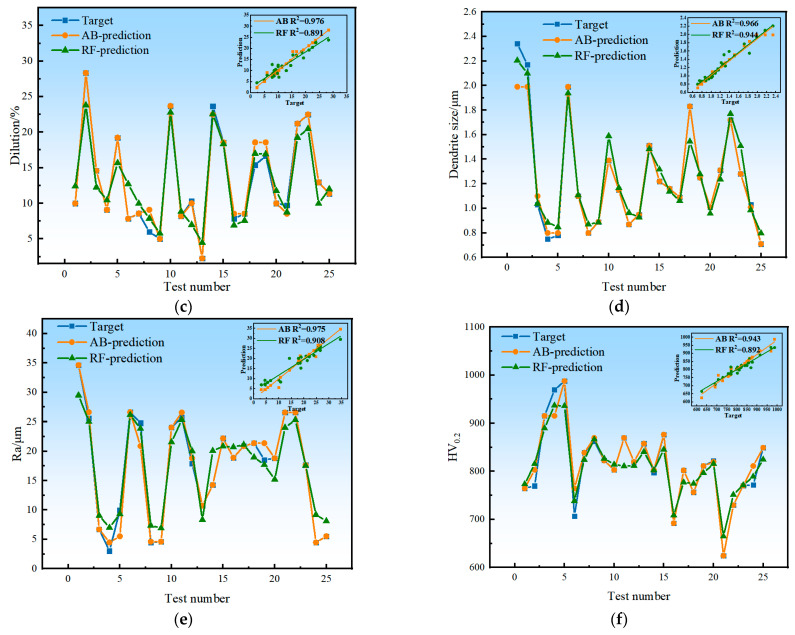
The experimental (blue marks), RF-estimated (green marks) and AB-estimated (orange marks) values for each of p = 25 experimental conditions: (**a**) cladding layer height; (**b**) molten pool depth; (**c**) dilution ratio; (**d**) grain size; (**e**) surface roughness; (**f**) microhardness.

The prediction model was constructed based on RF and AB algorithms. The process parameters such as laser power, scanning speed, overlap ratio and powder feeding rate were taken as the inputs, and the response values such as cladding layer height, molten pool depth, dilution ratio, grain size, surface roughness, and microhardness were taken as the outputs. After the model training was completed, the process parameters of the training group were again input into the model for prediction. The prediction performance of the two constructed prediction models was preliminarily verified, and their prediction accuracy was compared. The comparison between the predicted value and the real value of RF and AB models is shown in Figure 7. It can be seen that the AB model is more accurate than the RF model, and it also captures more volatile data points and shows greater sensitivity to abnormal data (maximum or minimum) in the prediction process of multiple response values. This makes the AB model more accurate in predicting the quality of high-speed laser cladding.

Then, the R^2^ values of the two models were obtained to check the fit between the prediction data and the measured data, as listed in Table 4. For all output responses, the closer the R^2^ values to 1, the higher the correlation between the predicted data and the measured data, and the better the model fit. By comparing the predicted values of the two models with the measured values in Figure 7 and Table 4, it can be found that the R^2^ value of the AB model is generally closer to 1 than that of the RF model.

The calculation method of R^2^ is shown in Equation (4):

(4) $$R^{2}=1-\frac{\sum_{i}{\left(\hat{y_{i}}-y_{i}\right)}^{2}}{\sum_{i}{\left(\bar{y_{i}}-y_{i}\right)}^{2}}$$

## 4. Importance Evaluation and Prediction Model Verification

### 4.1. Evaluation of the Importance of High-Speed Laser Cladding Process Parameters

Importance analysis is used to calculate the contribution of each process parameter to the target response value and rank the importance of process parameters. Based on this, the sensitivity relationship between each variable and the target value can be obtained, and the higher the ranking variable, the greater its impact on the target value. (i.e., target values are more sensitive to variables that rank higher). The feature selection process of machine learning (importance assessment) is integrated with the learner training process, and both are optimized within the same optimization process, i.e., the importance assessment was carried out automatically during the training of the learner.

**Figure 8:**
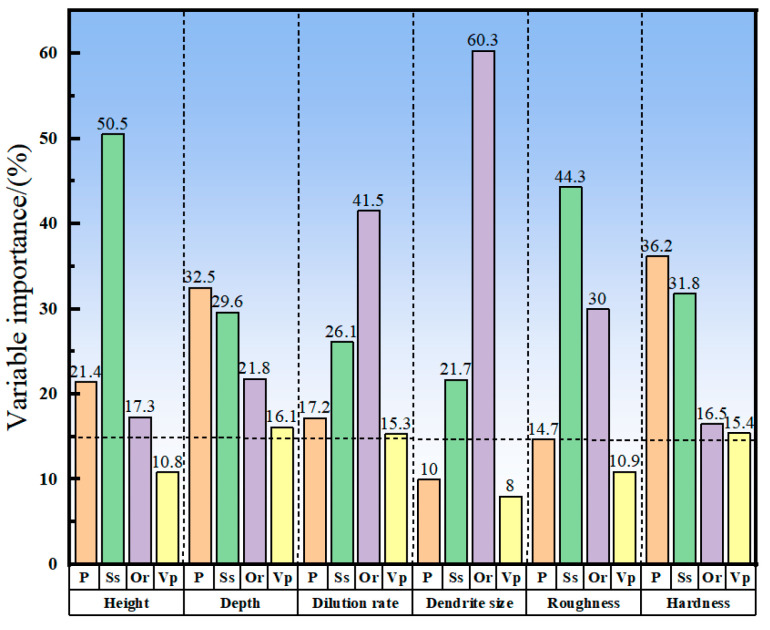
Variable importance measures of laser power, scan rate, overlap rate, and powder feeding rate.

The importance test of the AB prediction model was conducted. The response values of cladding layer height, molten pool depth, dilution ratio, grain size, surface roughness, and microhardness were used to evaluate the importance of laser power, scanning speed, overlap ratio, and powder feeding rate. The result is shown in Figure 8. Factors greater than 0.15 in importance were considered to have a great effect on response [19]. It can be seen that the change in laser power has the greatest effect on the microhardness of the coating and the molten pool depth, but it has little effect on the grain size. The variation in scanning speed and overlap ratio has a great effect on the coating quality. The scanning speed mainly affects the height and surface roughness of the cladding layer. Alongside that, the overlap ratio mainly affects the dilution ratio and surface roughness. The powder feeding rate has a relatively small effect on the coating quality, and it has the biggest effect on the molten pool depth. The results indicate that the densification of microstructure is significantly promoted by the increase in scanning speed, and the homogenization of dendrites near the surface is caused by the high overlap ratio.

On the other hand, it can be seen that when a higher coating thickness and a smaller surface roughness are required, adjusting the scanning speed is the most effective way to meet this requirement. To adjust the grain size near the surface of the coating and the dilution ratio of the coating, only the overlap ratio needs to be controlled. Additionally, coating depth and microhardness require a coordinated effect of power and scanning speed. These observations can provide a basis for adjusting process parameters in the subsequent process of cladding quality control.

### 4.2. Verification of High-Speed Laser Cladding Coating Quality Using the AB Prediction Model

**Figure 9:**
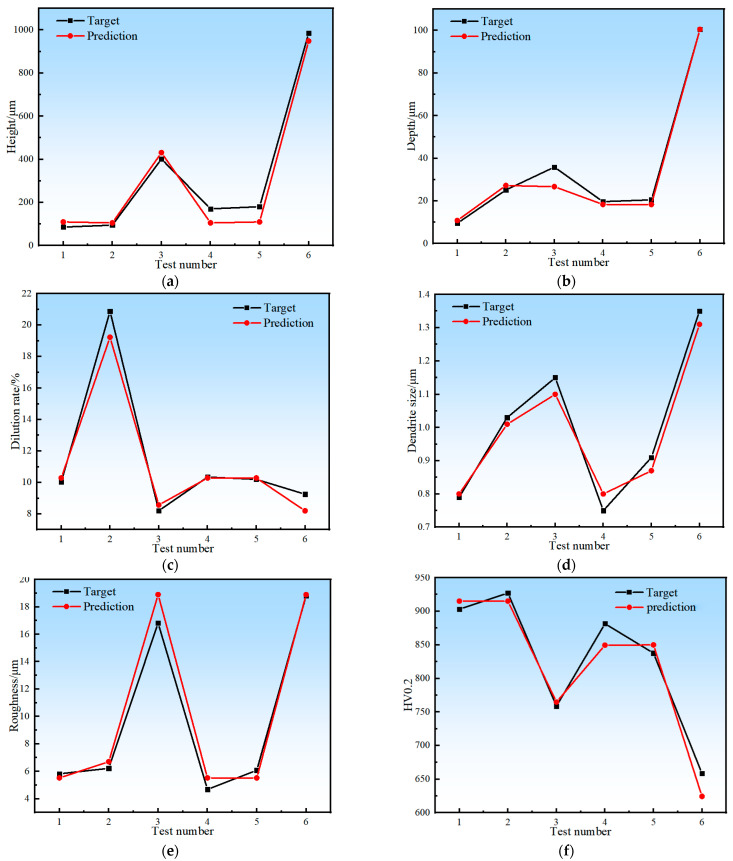
A fitting plot of the experimental values (black marks) and AB-estimated values (red marks) for the confirmatory experiments: (**a**) cladding layer height; (**b**) molten pool depth; (**c**) dilution ratio; (**d**) grain size; (**e**) surface roughness; (**f**) microhardness.

To test the predictive performance of the AB prediction model, another six sets of experiments were designed to compare and verify the response values and predicted values such as cladding layer height, molten pool depth, dilution ratio, grain size, and surface roughness and microhardness. The experimental design and results are listed in Table 5. The process parameters of the six validation experiments were selected based on the results of Taguchi’s experiments and were the optimal process parameters determined based on six indicators. The height of the cladding layer (H), depth of the melt pool (D), dilution ratio (η), grain size (Ds), surface roughness (Ra), microhardness (HV0.2), MAE and RMSE values were calculated to characterize the accuracy of the model. Specifically, MAE (mean absolute error) is the mean of the absolute error between the predicted and observed values; it indicates the degree of dispersion of the samples and is more sensitive to outliers. RMSE (root mean square error) is the sample standard deviation of the difference between the predicted and observed values. The smaller the values of MAE and RMSE, the higher the accuracy of the model. The calculation methods of MAE and RMSE are shown in Equations (5) and (6). The comparative verification results are illustrated in Figure 9. The precision characterization data of the AB model are listed in Table 5.

(5) $$\mathrm{MAE}=\frac{1}{25}\overset{25}{\underset{i=1}{\sum }}\left|\hat{y_{i}}-y_{i}\right|$$

(6) $$\mathrm{RMSE}=\sqrt{\frac{1}{25}\overset{25}{\underset{i=1}{\sum }}{\left(y_{i}-\hat{y_{i}}\right)}^{2}}$$

It can be seen from Table 6 that for the AB prediction model, the prediction error of each response value is less than 6%, indicating that there is a good correlation between the input parameters of the prediction model and the feature prediction results. According to the above prediction results, it is feasible to use the AB algorithm to build a high-speed laser cladding coating quality prediction model. It can play an important guiding role in predicting coating process parameters and even performance optimization.

## 5. Conclusions

In this study, two algorithms, RF and AB, were used to construct a coating quality prediction model. A total of 31 experiments were conducted in the training group and the verification group to train and verify the model, respectively. The conclusion of this study is as follows:

(1) The prediction process of high-speed laser cladding coating quality is complicated, and the RF and AB algorithms have strong mapping ability and nonlinear relationships. Therefore, when solving complex multivariate nonlinear problems, they can be adopted to address the issue of high-precision fitting, which is difficult for multiple-regression analysis, thereby achieving effective prediction of cladding quality. The AB prediction model captures volatile data points, and it is more accurate and more sensitive to abnormal data (maximum or minimum) than the RF in the prediction of multiple response values.
(2) The AB algorithm was also used to evaluate the importance of process parameters during the training of the learner. The most effective method to change the height and surface roughness of the cladding layer is to adjust the scanning speed. On the other hand, the overlap rate is the most important factor for controlling the dilution ratio and near-surface grain size of high-speed laser cladding. Alongside that, the microhardness of the coating and the thermal effect of the substrate can be effectively enhanced by adjusting the laser power and scanning speed. These observations provide a basis for the adjustment of process parameters in the stability control of the melting process.
(3) The experimental results indicate that the prediction errors of the AB model on the response values of cladding layer height, molten pool depth, dilution ratio, grain size, surface roughness and microhardness are all less than 6%. Therefore, the quality of the high-speed laser cladding layer can be predicted by this prediction model during machining. The prediction results indicate that the application of machine learning methods based on the AB algorithm has a certain reference value and practical significance in parameter prediction and performance optimization of the high-speed laser cladding process. It provides a new idea for the process parameter control in high-speed laser cladding.

## Figures and Tables

**Table 1:** The equipment parameters of the ZKZM-2000 fiber high-speed laser cladding system.

Parameter Type	Inversion
Power	500–2000 W
Wave length	1080 nm
Spot diameter	1.2 mm
Powder feeding method	Three-way coaxial powder feeding
Scanning speed	0–20 m
Gas flow	20–25 L/min
Maximum spindle speed	200 r/min
Machine stroke (*X*-axis)	3222 mm
Machine stroke (*Y*-axis)	400 mm
Machine stroke (*Z*-axis)	300 mm

**Table 2:** The chemical composition/Value (wt.%) of the Fe-Cr-Ni-based alloy powders.

**Chemical Composition**	C	Si	Cr	Ni	Mo	B	Fe
**Value (wt.%)**	0.15	4.5	22	13	2	1.6	Bal.

**Table 3:** Taguchi test factors and levels.

No.	Laser Power/	Scanning Speed/	Overlap Ratio/	Powder Flow Rate/
	P (W)	Ss (mm/min)	Or (%)	Vp (r/min)
1	660	3600	20	2.5
2	880	7200	35	3
3	1100	10,800	50	3.5
4	1320	14,400	65	4
5	1540	18,000	80	4.5

**Table 4:** The experimental design and the corresponding experimental responses.

No.	Process Parameter				Response					
	Laser Power/	Scanning Speed	Overlap Ratio	Powder Feeding Rate	Height	Depth	Dilution Rate	Grain Size	Roughness	Hardness
	P (W)	Ss (mm/min)	Or (%)	Vp (r/min)	H (μm)	D (μm)	H (%)	Ds (μm)	Ra (μm)	HV0.2
1	660	3600	20	2.5	245.15	27.24	10	2.34	34.63	764.83
2	660	7200	35	3	131.01	51.8	28.33	2.17	25.63	769.87
3	660	10,800	50	3.5	170.3	29.07	14.58	1.03	6.71	915.10
4	660	14,400	65	4	90.29	9.03	9.09	0.75	3.03	969.22
5	660	18,000	80	4.5	105.51	25.12	19.23	0.78	9.96	987.57
6	880	3600	35	3.5	431.22	36.7	7.84	1.99	26.63	707.07
7	880	7200	50	4	293.37	27.5	8.57	1.1	24.8	838.93
8	880	10,800	65	4.5	222.01	14.17	6	0.8	4.49	862.75
9	880	14,400	80	2.5	237.17	12.48	5	0.89	4.62	822.71
10	880	18,000	20	3	101.63	31.54	23.68	1.39	24.05	803.18
11	1100	3600	50	4.5	514.41	45.92	8.2	1.15	25.75	869.69
12	1100	7200	65	2.5	272.33	31.25	10.29	0.87	17.9	819.93
13	1100	10,800	80	3	467.05	10.86	2.27	0.95	10.75	857.91
14	1100	14,400	20	3.5	109.78	34.1	23.68	1.51	14.26	797.77
15	1100	18,000	35	4	72.38	16.51	18.57	1.22	22.2	876.60
16	1320	3600	65	3	755.01	64.45	7.87	1.16	18.9	692.25
17	1320	7200	80	3.5	340.56	31.79	8.54	1.09	20.89	802.23
18	1320	10,800	20	4	184.97	33.63	15.38	1.83	21.38	756.58
19	1320	14,400	35	4.5	137.86	27.57	16.67	1.25	18.55	811.26
20	1320	18,000	50	2.5	165.23	18.36	10	1.01	18.82	822.14
21	1540	3600	80	4	948.57	102.4	9.75	1.31	26.57	624.48
22	1540	7200	20	4.5	190.51	51.29	21.21	1.72	26.57	729.75
23	1540	10,800	35	2.5	226.66	65.8	22.5	1.28	17.65	770.49
24	1540	14,400	50	3	208.09	31	12.96	1.03	4.49	771.56
25	1540	18,000	65	3.5	171.21	21.95	11.36	0.71	5.53	849.48

**Table 5:** The experimental design of the AB model and the corresponding experimental responses.

No.	Process Parameter				Response					
	Laser Power/	Scanning Speed	Overlap Ratio	Powder Feeding Rate	Height	Depth	Dilution Rate	Grain Size	Roughness	Hardness
	P (W)	Ss (mm/min)	Or (%)	Vp (r/min)	H (μm)	D (μm)	H (%)	Ds (μm)	Ra (μm)	HV0.2
1	660	14,400	65	3.5	85.78	9.57	10.04	0.79	5.83	903.29
2	660	18,000	50	4.5	95.35	25.15	20.87	1.03	6.23	927.11
3	880	3600	65	2.5	401.57	35.9	8.21	1.15	16.82	758.64
4	880	18,000	65	3.5	170.66	19.7	10.35	0.75	4.69	881.57
5	1100	18,000	65	3.5	180.59	20.51	10.20	0.91	6.09	837.64
6	1540	3600	80	3	985.15	100.5	9.25	1.35	18.82	658.49

**Table 6:** Characterization of precision data of the AdaBoost model.

Model	Height (H)			Depth (D)			Dilution (η)		
	MAE	RMSE	R^2^	MAE	RMSE	R^2^	MAE	RMSE	R^2^
AdaBoost	39.372	45.009	0.979	1.603	1.678	0.997	0.578	0.819	0.963
**Model**	**Dendrite size (Ds)**			**Roughness (Ra)**			**Hardness (Hv0.2)**		
	**MAE**	**RMSE**	**R^2^**	**MAE**	**RMSE**	**R^2^**	**MAE**	**RMSE**	**R^2^**
AdaBoost	0.035	0.038	0.966	0.723	0.972	0.971	17.992	21.011	0.949

## Data Availability

There are no additional data reported in this study to support the results.
